# Mechanism of antiphospholipid antibody-mediated thrombosis in antiphospholipid syndrome

**DOI:** 10.3389/fimmu.2025.1527554

**Published:** 2025-03-13

**Authors:** Leiyi Yang, Ruibing Guo, Hongjiang Liu, Bo Chen, Changpei Li, Ruiting Liu, Shuyi Liao, Qibing Xie, Geng Yin

**Affiliations:** ^1^ Department of Rheumatology and Immunology, West China Hospital, Sichuan University, Chengdu, China; ^2^ Health Management Center, General Practice Medical Center, West China Hospital, Sichuan University, Chengdu, China

**Keywords:** antiphospholipid syndrome, antiphospholipid antibody, β2-glycoprotein I, thrombotic event, two hit model

## Abstract

Antiphospholipid syndrome (APS) is an autoimmune disease characterized by the occurrence of thrombotic or obstetrical events in patients with persistent antiphospholipid antibodies (aPL). Thrombotic events, the primary pathological hallmarks and clinical manifestations, are among the leading causes of mortality in APS. Our understanding of the mechanism underlying APS-related thrombosis has significantly advanced in recent years. The presence of aPL, particularly anti-β2-glycoprotein I (anti-β2GPI) antibodies, is a major driver of thrombosis. The proposed pathophysiological mechanisms of aPL-mediated pro-thrombotic events can be broadly categorized into three types: disruption of anticoagulant reactions and fibrinolysis, interference with coagulation cascade cells, and complement activation. A triggering ‘second hit’ is typically necessary to initiate thrombosis. The development of animal models of APS has further refined our understanding of the role of aPL in thrombosis. In this review, we focused on the role of β2GPI-dependent aPL in thrombosis of thrombotic APS.

## 1 Introduction

Antiphospholipid syndrome (APS), first described in the early 1980s (1), is defined by the presence of antiphospholipid antibodies (aPL) in patients with thrombotic complications and/or adverse pregnancy outcomes (2). APS is one of the most common causes of acquired thrombophilia, affecting both arterial and venous circulation, particularly in young people (3, 4). Thrombotic events are the most frequent clinical manifestations and the leading causes of mortality in APS (4). The annual incidence and prevalence of APS in adults are 1-2 and 40-50 per 100,000 individuals, respectively (5). The 10-year survival rate for patients with APS has been reported to be 90.7% (4). Less than 1% of APS patients may develop catastrophic APS, characterized by life-threatening microvascular thrombosis in at least three organs, with a mortality rate of up to 50% (6), typically occurring within one week (7). Emerging evidence highlights significant sex-related differences in APS pathogenesis, with a striking 5:1 female predominance (8). In thrombotic APS, women tend to present venous thrombosis at a younger age while men manifest arterial events later in life (9). This happens because estrogen and progesterone work together in specific ways. First, estrogen induces a prothrombotic environment through various effects on the hemostatic pathways (10). Second, progesterone activates platelets through glucocorticoid receptor signaling, increasing the likelihood of venous thrombosis when progesterone levels are high (11). Women with APS are at substantially elevated risk for pregnancy-related complications, with pooled analyses demonstrating 12.3-fold increased odds of severe preeclampsia, 9.1-fold risk of fetal loss, and 6.8-fold higher perinatal mortality compared to the general obstetric population (12).

APS can present either as a primary condition (primary APS) (13) or in association with other systemic autoimmune diseases, most notably systemic lupus erythematosus (SLE), in which case it sometimes referred to as secondary APS (8, 14). However, the expert committee on APS advises against using the term ‘secondary APS’ (2). This is primarily because there are no significant differences in the clinical consequences between primary APS and so-called secondary APS (14, 15). The 2023 ACR/EULAR classification criteria emphasize a phenotype-based classification framework over etiological associations (16). It is now generally accepted that APS manifests in two main clinical variants: thrombotic APS and obstetric APS (17, 18). In addition to the presence of aPL, thrombotic APS is characterized by clinical features related to venous, arterial, or microvascular thrombosis, while obstetric APS is distinguished by pregnancy complications (17, 19). Over the past 30 years, our understanding of the mechanism of APS thrombosis has evolved significantly. However, the exact mechanisms are still not fully understood, which may explain why current treatment relies mainly on anticoagulants (17). Although anticoagulants are somewhat effective in preventing aPL-associated thrombosis (especially venous), they exhibit limited therapeutic impact on the microvascular manifestations of APS that affect the heart, kidneys, skin, and brain (20). Despite existing treatments, mainly including oral anticoagulants and/or anti-aggregation agents, patients with APS continue to experience considerable morbidity and mortality (4). Therefore, it is imperative to intensify efforts to develop therapeutic strategies to prevent these critical complications. Recently, Meroni et al. proposed that thrombotic and obstetric APS may represent two distinct diseases mediated by the same antibodies (18).

The aPL, including anticardiolipin (aCL), lupus anticoagulant (LA) and anti-β2-glycoprotein (anti-β2GPI) antibodies, recognize plasma proteins that bind avidly to anionic phospholipid surfaces, with β2GPI being the primary target (2, 21). While there is general consensus that aPL detected in patients with APS mediate thrombosis, the underlying pathophysiological mechanisms remain under debate (17, 22–24). Herein, we review current evidence on the role of β2GPI-dependent aPL in thrombosis of thrombotic APS. Our aim is to enhance understanding of these mechanisms, potentially illuminating future therapeutic targets and strategies to prevent thrombotic events.

## 2 Murine models of APS thrombosis

Animal models are crucial for investigating the mechanisms of aPL-induced thrombogenesis. In the 1990s, Pierangeli et al. developed the femoral vein pinch model, a mouse model for studying aPL-induced venous thrombosis *in vivo* (25–27). In this model, the appropriate dose of IgG-APS was injected intraperitoneally (i.p.) at 0 and 48h. The surgical procedure was performed 72 hours after the first injection. The model enabled the study of thrombus size and growth dynamics in the vein using a transilluminator equipped with digital video analysis (25–27). Several research groups have adopted this mouse model to investigate mechanisms of aPL-mediated thrombosis (28–31). Then, Jankowski’s laboratory adapted a murine model of arterial thrombosis induced by a photochemical reaction (32, 33). In this model, thrombosis was induced in the left carotid artery using filtered green light irradiation combined with the fluorescent dye rose-bengal in mice or hamsters (32, 33). Purified human β2GPI mAbs or IgG from APS patients were infused 15 minutes prior to photochemical vessel injury. Thrombus formation was continuously monitored by a transilluminator mounted on the artery and quantitatively analyzed with image processing software (32, 33). Furie and colleagues later utilized a laser-induced arteriole injury model in mice and applied intravital microscopy to image thrombus formation in real time within the microcirculation (34, 35). Using the laser-induced thrombosis model and intravital microscopy, the same group investigated the *in vivo* roles of platelets and endothelial cells in anti-β2GPI antibody/β2GPI complex-mediated thrombosis (36, 37). Seshan et al. developed a mouse model of thrombotic microangiopathy that replicated the early-stage pathophysiology of thrombotic microangiopathy induced by aPL in APS patients (38). This model used smaller amounts of aPL-IgG and enabled the investigation of mechanisms underlying renal vascular thrombosis (38). In addition, two research groups employed a ferric chloride-induced thrombosis model to explore the molecular and cellular events mediated by aPL in vascular thrombosis *in vivo* (39, 40).

Deep vein thrombosis represents the most frequently occurring form of thrombosis in APS (4). Manukyan and colleagues recently introduced a novel mouse model of flow restriction-induced thrombosis, termed stenosis, which simulates the clinical features of deep vein thrombosis in humans (41, 42). In this model, a spacer was positioned around the exposed inferior vena cava (IVC), and a permanent ligature was tightened below the left renal vein to create narrowing. The wire was then removed to prevent complete vessel occlusion. Antibodies were administered one hour before inducing flow restriction (41). Building on this thrombosis model, Knight’s group developed a murine model of APS-induced thrombosis via flow restriction or stenosis of the IVC (43, 44). However, most existing murine models of APS-related thrombosis have been confined to microscopic vascular beds. To better replicate large-vein thrombosis, Knight and colleagues recently developed an electrolytic IVC model of aPL-accelerated thrombosis (45, 46). A 30-gauge silver-coated copper wire was attached to a 25-gauge needle, which was inserted into the exposed IVC. A mild direct current of 250 μA was applied for 15 minutes. The mouse was then treated with 500 μg of aPL-IgG and allowed to recover. Thrombus size and content were assessed after 24 hours (46).

Patients with APS continuously produce aPL, leading to vascular thrombosis in both small and large vessels across venous and arterial beds (17, 47). However, existing animal models have demonstrated that a single, temporary aPL injection is insufficient to induce a pro-thrombotic phenotype. These models are often restricted to studying thrombosis in only one type of blood vessel. Furthermore, the aPL dosage, injection intervals, and choice of stimulating factor vary widely among these models.

## 3 Antiphospholipid antibodies and β2-glycoprotein I

The aPL included in the 2006 Sydney classification criteria are LA, IgG and/or IgM aCL antibodies, and anti-β2GPI antibody of IgG and/or IgM isotype (2). A diagnosis of APS requires persistently positive aPL (detected on two occasions ≥12 weeks apart) and at least one of two clinical manifestations: vascular thrombosis or pregnancy morbidity (2, 16). However, some individuals exhibit a clinical profile highly suggestive of APS but consistently test negative for ‘criteria’ aPL (48). The term ‘seronegative APS’ has been proposed to describe this subset of patients (49, 50). Seronegative APS patients are not entirely without autoantibodies but instead present with ‘extra-criteria’ autoantibodies. Notably, the clinical validity of these non-criteria antibodies varies significantly: while anti-phosphatidylserine/prothrombin (aPS/PT) and anti-β2GPI-domain I antibodies show strong mechanistic evidence and are under consideration for inclusion in future classification criteria (51–53), others such as anti-annexin antibodies and anti-phosphatidylethanolamine lack robust clinical validation (51). It is likely that all subpopulations of ‘criteria’ or ‘extra-criteria’ autoantibodies in patients with APS can influence their hemostatic balance. Nonetheless, it is widely accepted that APS-related thrombosis is driven by aPL, with antibodies against β2GPI being the most significant (17).

Initially, it was thought that aPL could directly recognize anionic phospholipids. However, some studies conducted in the 1990s confirmed that these antibodies do not bind phospholipids directly but instead interact with phospholipids via the plasma protein β2GPI (21, 54, 55). In APS, phospholipid-bound β2GPI is the primary target of aPL, and anti-β2GPI antibodies are thought to play a central role in the mechanisms of thrombosis (17, 24, 56). Multiple studies using animal models have demonstrated that anti-β2GPI antibodies or β2GPI-dependent antibodies play important roles in inducing thrombosis (33, 41, 57). Further research, which separated the heterogeneous aPL population from APS patients into different subpopulations, showed that anti-β2GPI antibodies significantly enhanced the thrombotic response in a mouse model (36).

β2GPI, also known as apolipoprotein H, is a 50-kDa phospholipid-binding glycoprotein present in plasma at a concentration of approximately 200 μg/mL (58). The primary function of β2GPI remains largely unknown, although it has been reported to have roles in anticoagulant activity, antiangiogenic activity, complement regulation, and other physiological process (59–62). β2GPI exists in several conformations, including J-elongated, S-twisted, and O-circular, with the J conformation likely being predominant under physiological conditions (63). Normally, β2GPI circulates in a circular form, but in the presence of elevated aPL or exposed anionic phospholipids on cell membranes, it adopts an open conformation, which may contribute to the pathogenesis of APS and thrombosis (62). However, the mechanisms underlying these conformational changes remain unclear. Structurally, β2GPI is composed of 326 amino acids arranged into five homologous domains, with domain V containing a unique lysine cluster and a C-terminal loop (64–66). The specific structure of domain V forms a binding site for negatively charged phospholipids, such as cardiolipin and phosphatidylserine (67, 68). When β2GPI binds to the surface of anionic phospholipids, it exposes a hidden epitope that is recognized by aPL in APS (69–71). These aPL do not recognize β2GPI in solution and only bind to domain I of β2GPI which has undergone a conformational change (70, 71).

## 4 Potential mechanisms of aPL-mediated thrombosis

The possible pathogenesis of thrombosis mediated by β2GPI-dependent aPL includes (1) disruption of fluid-phase coagulation by interfering with protein C, antithrombin, annexin A5, and fibrinolysis (2), impairment of coagulation cascade cell functions by interacting with monocytes, endothelial cells, neutrophils, and platelets (3), and complement activation. It is now widely accepted that a ‘second hit’ is necessary to trigger thrombotic events (17, 47, 72, 73).

### 4.1 Disruptions of fluid-phase coagulation

#### 4.1.1 Inhibition of the protein C pathway

Protein C is an important vitamin K-dependent anticoagulant that becomes activated when thrombin binds to thrombomodulin. Activated protein C (APC) plays crucial anticoagulant and antithrombotic roles by binding to and inactivating the procoagulant factors Va and VIIIa (74). Researchers discovered that the activation of protein C and the function of APC are inhibited by purified immunoglobulin fractions from patients with APS (75, 76). APL can disrupt the protein C system in several ways, including inhibiting the assembly of the protein C complex, interfering with protein C activation, and blocking thrombin formation (77–79). Murine monoclonal anti-β2GPI antibodies, in the presence of β2GPI, have been demonstrated to inhibit the anticoagulant activity of APC *in vitro* (80). In addition, anti-β2GPI antibodies and β2GPI-dependent LA can induce APC resistance, increasing the risk of venous thromboembolism in patients with APS (81, 82). These studies indicate that protein C dysfunction caused by aPL is primarily mediated by β2GPI. Autoantibodies directed against protein C have been detected in the serum APS patients, and they show a significant correlation with APC resistance and thrombosis in these individuals (83).

#### 4.1.2 Inhibition of antithrombin activity

Antithrombin is the primary inhibitor of thrombin, as well as factors IXa and Xa. As early as the 1980s, it was reported that an APS patient with recurrent thrombosis had normal levels of antithrombin antigen but reduced functional activity (84). APL can inhibit the cofactor activity of the heparin/antithrombin III complex and interfere with the formation of antithrombin III-thrombin complexes, thereby promoting thrombosis in patients with APS (85, 86). Two studies have demonstrated that injecting anti-prothrombin autoantibodies into mice can induce a pro-thrombotic phenotype, which may be linked to the inhibition of antithrombin activity (86, 87).

#### 4.1.3 Disruption of annexin A5 anticoagulant shield

Annexin A5 is a protein that binds to anionic phospholipids with high affinity. It forms a protective crystal shield on vascular cells, inhibiting phospholipid-dependent coagulation reactions. Rand et al. were the first to report that aPL reduce the levels of annexin A5 and promote plasma coagulation on vascular endothelial cells. This finding suggested a potential mechanism for thrombosis (88). They hypothesized that aPL might increase resistance to the anticoagulant effects of annexin A5. Based on this hypothesis, Rand and colleagues developed a method to detect what they termed ‘annexin A5 resistance’ in plasma, which they confirmed in several populations of patients with APS (89). Subsequently, an *in vitro* study by the same group showed that anti-β2GPI antibodies, in complex with β2GPI, can disrupt annexin A5’s anticoagulant shield (90). This disruption exposes procoagulant phosphatidylserine, increasing the risk of thrombosis. Hydroxychloroquine has been found to inhibit the ability of β2GPI immune complexes to disrupt the protective annexin A5 barrier on the surface of vascular endothelial cells (91). This provides new evidence for the therapeutic potential of this old antimalarial drug in APS patients.

#### 4.1.4 Insufficient fibrinolysis

Fibrinolysis is the process by which fibrin, formed during blood coagulation, is broken down and liquefied. Fibrinolysis is a crucial anticoagulant process *in vivo*. Musiał J et al. discovered that fibrin clots in thrombotic APS patients are composed of thin fibers and small pores. This structure makes the clots firmer, quicker to form, and slower to degrade compared to those in similar VTE cases (92). The altered fibrin structure is more resistant to lysis, which negatively impacts anticoagulation. Annexin A2 acts as a receptor for β2GPI and tissue plasminogen activator (tPA), playing a significant role in the process of fibrinolysis (93). Studies have demonstrated that patients with APS have autoantibodies against the Annexin A2, with high titers of these antibodies being significantly correlated with thrombosis (94, 95), as they inhibit tPA-dependent plasmin generation. Additionally, anti-β2GPI antibodies in APS patients can neutralize the capacity of β2GPI to enhance tPA activity, thereby further inhibiting fibrinolysis (96).

### 4.2 Cell-mediated events

**Figure 1:**
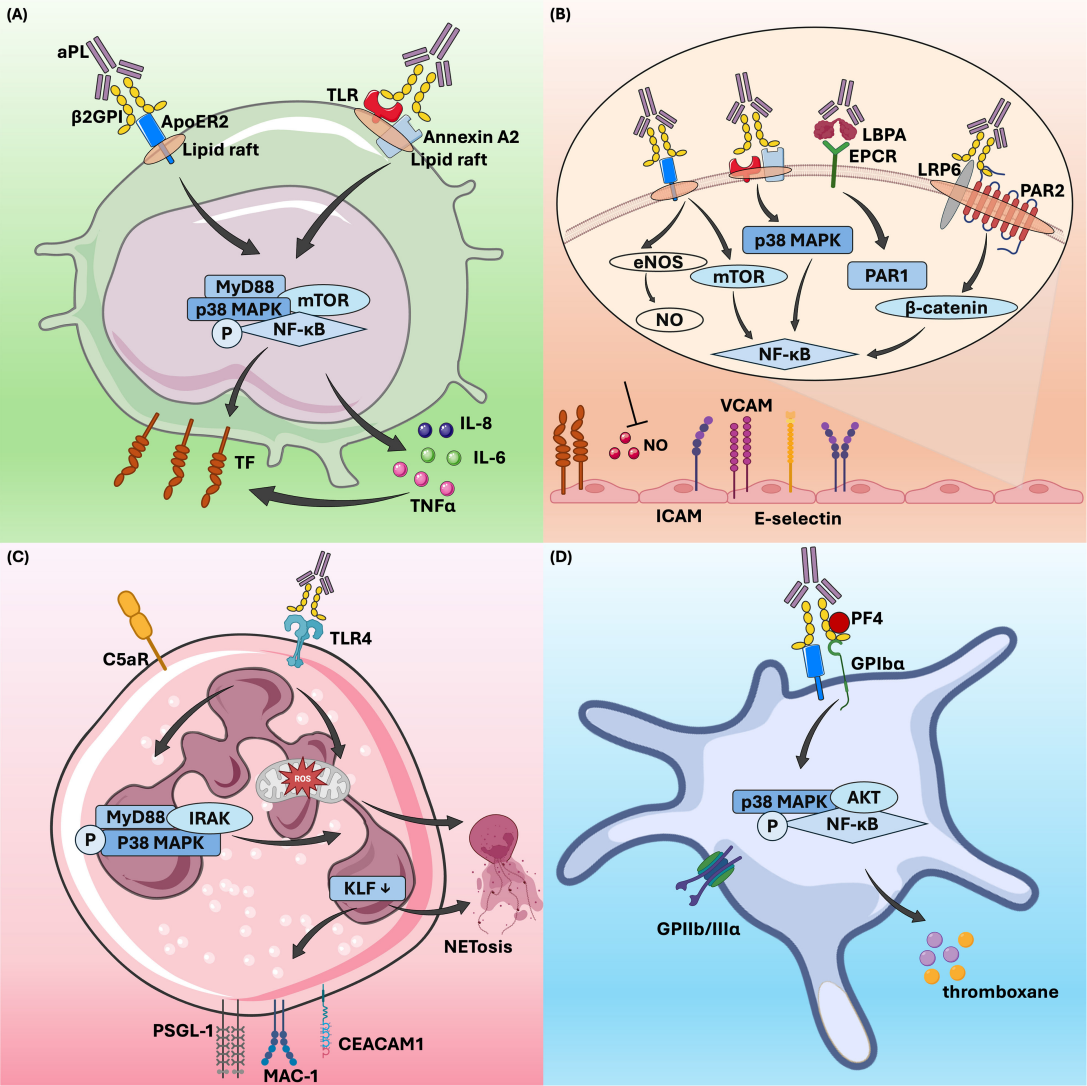
β2GPI-dependent aPL effects on cells in thrombotic antiphospholipid syndrome. β2GPI-dependent aPL activates monocytes **(A)**, endothelial cells **(B)**, neutrophils **(C)**, and platelets **(D)** through various signaling pathways, thereby regulating downstream cellular activities and contributing to thrombosis in APS. aPL, antiphospholipid antibody;β2GPI, β2-glycoprotein I; ApoER2, apolipoprotein E receptor 2; TLR, toll-like receptor; MyD88, myeloid differentiation primary response gene 88; MAPK, mitogen-activated protein kinase; mTOR, mammalian target of rapamycin; NF-κB, nuclear factor kappa B; AKT, protein kinase B; TF, tissue factor; TNFα, tumor necrosis factor-alpha; LBPA, lysobisphosphatidic acid; EPCR, endothelial protein C receptor; LRP6, LDL receptor-related protein 6; PAR1, protease-activated receptor 1;eNOS, endothelial nitric oxide synthase; ICAM, intercellular adhesion molecule; VCAM, vascular cell adhesion molecule; ROS, reactive oxygen species; NETs, neutrophil extracellular traps; KLF, krüppel-like factor 2; PSGL-1, P-selectin glycoprotein ligand 1; IRAK, interleukin-1 receptor-associated kinase; MAC-1, macrophage-1 antigen; CEACAM1, carcinoembryonic antigen related cell adhesion molecule 1; PF4, platelet factor 4; GPIIbIIIα, glycoprotein IIIbIIIα.

Vascular inflammation is another critical mechanism of thrombosis in APS. Various types of cells are involved in the inflammatory process, including endothelial cells, monocytes, and neutrophils. Anti-β2GPI antibodies bind to membrane-bound β2GPI, triggering intracellular signaling and thereby promoting inflammation (**Figure 1**).

#### 4.2.1 On endothelial cells: expression of adhesion molecules and procoagulant substances

Quiescent endothelial cells are crucial for maintaining blood flow by expressing anticoagulant proteins and generating an actively antithrombotic surface within blood vessels (97). However, when endothelial cells are disrupted, their membranes can shift from an anticoagulant surface to a procoagulant phenotype. This change is mainly characterized by the induction of tissue factor (TF), plasminogen activator inhibitors, and the synthesis of specific binding sites for coagulation factors (97). TF is a single-stranded transmembrane glycoprotein that acts as a key initiator of the blood coagulation cascade by binding to factor VIIa (98, 99). Under normal conditions, TF is not expressed by intravascular cells but can be induced in monocytes and endothelial cells in response to nonphysiological or pathophysiological stimulation (100, 101). Numerous studies have shown that aPL, especially anti-β2GPI antibodies, can activate endothelial cells, promoting thrombosis in APS (102–104). Endothelial cells activated by aPL display increased expression of adhesion molecules, such as E-selectin, vascular cell adhesion molecule-1, and intercellular adhesion molecule-1 (102, 105).

The induction of TF expression in endothelial cells by antiphospholipid sera was reported in 1993 (106). Subsequent *in vitro* studies have confirmed that aPL can induce the expression of TF in endothelial cells (107, 108). Additionally, aPL-activated endothelial cells contribute to a prothrombotic state through mechanisms such as releasing microparticles with proinflammatory and procoagulant properties (109, 110), producing proinflammatory cytokines (111), and reducing levels of endothelial cell-derived nitric oxide (112).

There are multiple pathways through which activated endothelial cells mediate the prethrombotic state in APS. Currently, it is accepted that the binding of β2GPI -antibody complexes to Annexin A2 or toll-like receptor (TLR) on endothelial cell surfaces triggers the activation of the p38 mitogen-activated protein kinase (MAPK) and nuclear factor kappa B (NF-κB) signaling pathways, which in turn promote the expression of procoagulant substances (29, 113, 114). Annexin A2 has been identified as a significant aPL receptor on endothelial cell membranes and is essential in endothelial cell activation and thrombosis in APS (95, 115). Research suggested that Annexin A2 might be part of a larger aPL receptor complex on endothelial cells, potentially forming co-receptors with TLR2 or TLR4 to mediate this activation (116, 117). In contrast, Annexin V, another member of the annexin family, acts protectively by blocking aPL from binding to phospholipids on the cell membrane. These antibodies cannot interact with endothelial cells unless Annexin V is interrupted (118). Endothelial protein C receptor (EPCR) serves as a receptor for β2GPI/anti-β2GPI antibody complexes in APS, playing crucial roles in anticoagulation and placental development. In APS, anti-EPCR antibodies can inhibit protein C activation, thereby increasing the risk of fetal loss and thrombotic events (119, 120). Binding of aPL to EPCR accelerates the endocytosis of the EPCR- lysobisphosphatidic acid complex, which leads to thrombin-induced and protease-activated receptor 1-mediated endothelial cell activation (121, 122). Furthermore, different EPCR haplotypes, particularly the H1 haplotype, can influence APS symptoms, modulating the risk of arterial thrombosis (123). Apolipoprotein E receptor 2 (ApoER2), also known as LDL receptor-related protein 8 (LRP8), is a member of the low-density lipoprotein receptor family and plays a crucial role in the pathogenic mechanism of aPL. Compared to ApoER2+/+ mice, ApoER2-/- mice exhibited prolonged vascular occlusion times induced by aPL, along with significant reductions in aPL-induced vascular TF activity, thrombosis formation, and monocyte activation (30). ApoER2 also participates in the inhibition of endothelial cell migration and regeneration by aPL. In cell experiments conducted by Ulrich V. et al., siRNA knockdown of ApoER2 in endothelial cells restored migration capacity, which had been suppressed by aPL (124). In APS, ApoER2 facilitates thrombosis by serving as a scaffold for the assembly of protein phosphatase 2A, leading to antagonism of endothelial nitric oxide synthase (eNOS) (125). This mechanism involves the recruitment of Disabled-2 and Src homology domain-containing transforming protein 1 to ApoER2, which activates protein phosphatase 2A and promotes the dephosphorylation of protein kinase B (AKT) and eNOS, ultimately contributing to aPL-induced thrombosis (40).

#### 4.2.2 On monocytes: induction of tissue factor

Monocytes contribute to thrombosis in APS primarily through the expression of TF (126). Kornberg et al. demonstrated that murine monoclonal aCL can induce TF expression in monocytes and enhance TF procoagulant activity (127). Later, Guadrado et al. found that monocytes from APS patients with thrombosis showed significantly increased TF mRNA expression and TF-related procoagulant activity compared to those without thrombosis (128). Subsequent studies confirmed that human monoclonal aCL and IgG from patients with APS promote TF expression and boost TF activity in monocytes (129, 130). Extensive research has since established that aPL, especially anti-β2GPI antibodies, can induce TF expression on monocytes when they form β2GPI/anti-β2GPI immune complexes (131–134). These complexes interact with various cell surface receptors, such as phosphatidylserine, ApoER2, and Annexin A2, as well as coreceptors like TLRs (135). These interactions activate signaling pathways, including mitogen-activated protein kinase kinase 1 (MEK-1)/extracellular regulated protein kinases (ERK), p38 MAPK, mammalian target of rapamycin (mTOR), and NF-κB, predominantly through the TLR4-myeloid differentiation primary response 88 (MyD88) pathway (131–134, 136–138). Furthermore, the β2GPI/anti-β2GPI complexes upregulate inflammatory cytokines through the TLR/MyD88 and NF-κB pathways, leading to increase TF expression in monocytes (134, 139). *In vitro* studies demonstrated that monocytes from healthy donors incubated with monoclonal aPL or affinity-purified anti-β2GPI antibodies showed significantly higher secretion of tumor necrosis factor-alpha (TNFα) compared to those incubated with control IgG, further amplifying TF expression (134, 140, 141).

The expression of TF is also regulated by tissue factor pathway inhibitor (TFPI), which inhibits factors VIIa and Xa. One study revealed that anti-β2GPI antibodies suppress TFPI activity, thereby enhancing factor Xa generation (142). Notably, recent findings showed that aPL dissociated TFPI from monocytes, increasing the risk of thrombosis in APS patients (142). Furthermore, protease-activated receptors (PARs), which are triggered by thrombin or factor X, contribute to the production of pro-inflammatory cytokine (135). It was reported that the expression of PARs was elevated in monocytes from APS patients, and inhibition of PAR2 prevents aPL-induced TF expression (143).

#### 4.2.3 On neutrophil: increased release of neutrophil extracellular traps

Neutrophils are the most abundant leukocyte subsets in human peripheral blood and play an important role in innate immune against the invasion of various pathogenic microbes (144). They defend against external pathogens through multiple mechanisms, including the release of neutrophil extracellular traps (NETs), which are meshwork substances composed of DNA, histones, and antibacterial proteins (145). The release of NETs is the most striking phase in a unique cell death process called NETosis, distinct from apoptosis and necrosis (146). NET formation is a double-edged sword, playing a role in the pathogenesis of inflammatory and autoimmune disorders (147, 148).

Compared to the extensive research on the aforementioned cell types, the interactions between aPL and neutrophils have been less thoroughly investigated. Initially, only two studies demonstrated that aPL could directly activate neutrophils (149, 150), a process that may be amplified by complement C5, thereby promoting blood coagulation (151). With the growing recognition of the connection between NETs and thrombosis in non-autoimmune contexts, increasing attention has been given to the role of neutrophils in APS-related thrombosis (152, 153). Leffler et al. discovered that APS sera exhibited an impaired ability to degrade NETs, a dysfunction associated with specific clinical features (154). In recent years, Knight’s group has conducted several pivotal studies examining the role of neutrophils in APS-related thrombosis. In a 2015 study, they identified elevated levels of NETs in the circulation of APS patients and demonstrated that anti-β2GPI IgG could promote NET release *in vitro* (155). Moreover, these aPL-stimulated NETs were shown to facilitate thrombin generation *in vitro* (155), introducing a novel mechanism of thrombosis in patients with APS (156). Knight and his team later confirmed the *in vivo* relevance of NETs in thrombosis using a mouse model of APS. In this model, they observed that APS-associated thrombi were enriched with NETs and that NET-disrupting treatments could prevent APS IgG-mediated thrombosis (43). To further understand the mechanism of neutrophil hyperactivity in APS, they conducted a transcriptome analysis of APS neutrophils. This analysis revealed a proinflammatory gene expression signature, with overexpressed genes linked to interferon signaling, cellular defense, and intercellular adhesion (44). Among the upregulated genes, they identified a notable group of leukocyte immunoglobulin-like receptor (LILR) genes, which included all activating members of the LILR family: LILRA1, LILRA2, LILRA3, LILRA4, LILRA5, and LILRA6 (44). The role of these LILR members in regulating thromboinflammation in APS remains unclear. More recently, Knight’s group has showed that neutrophils in APS exhibit increased adhesive potential, lowering the threshold for NETosis and elevating the risk of thrombotic events (157). Additionally, a specific neutrophil subgroup known as low-density granulocytes, which is elevated in APS, exhibits a higher propensity for NET release (158). Mechanistically, anti-β2GPI antibodies promote NETs formation in a time- and concentration-dependent manner by forming complexes with β2GPI, activating TLR4, triggering ROS production, and inducing NET release via the MyD88-IRAKs-MAPKs pathway (159). Targeting NET release, for instance, by activating adenosine receptor, has been shown to reduce thrombosis in APS mouse models (46).

In APS, neutrophils exhibit an increased tendency to interact with endothelial cells, driven by the upregulation of adhesion molecules induced by aPL. The loss of the transcription factor Krüppel-like factor 2 (KLF2) is a key factor that transforms neutrophils into a prothrombotic state, enhancing their migration, adhesion, and release of factors like P-selectin glycoprotein ligand 1 (PSGL-1), which facilitates binding to the endothelium (160). Neutrophils in APS exhibit lower KLF2 levels, resulting in increased clustering of PSGL-1, elevated NET formation, and higher TF activity. Additionally, these neutrophils also upregulate other adhesion proteins, including CD64, carcinoembryonic antigen related cell adhesion molecule-1, β2-glycoprotein, and activated macrophage-1 antigen, all of which contribute to their heightened thrombotic potential (157).

#### 4.2.4 On platelets: enhanced platelet activation/aggregation

Platelets are essential for physiological hemostasis, as they aggregate and adhere to injured vessel walls, become activated, and release granule, promoting blood clotting and thrombus formation. Thrombocytopenia is a common clinical feature in patients with APS, leading to the hypothesis that aPL may bind to platelets, causing aggregation and thrombosis. The proposed causes of thrombocytopenia include platelet destruction by autoantibodies targeting platelet glycoproteins, as well as platelet activation and depletion initiated by aPL (161). Studies have confirmed that platelet activation in patients with APS is primarily linked to β2GPI-dependent antiphospholipid antibodies (33, 162, 163). The binding of anti-β2GP auto-antibody/β2GPI immune complex to ApoER2 and glycoprotein Ibα can promote platelet activation (164–167). These receptors cross-link with anti-β2GPI, activating platelets and promoting the release of thromboxane A2. This process neutralizes the inhibitory effect of β2GPI on von Willebrand factor, thereby enhancing platelet adhesion and aggregation (166–168). Platelet factor 4 (PF4) is a specific protein released by activated platelets that promotes thrombosis. In patients with APS, β2GPI can form a stable complex with PF4. The binding of anti-β2GPI antibody to the complex triggers p38 MAPK phosphorylation and induces the production of thromboxane B2 (169). In a mouse model using fluorescently labeled β2GPI and anti-β2GPI autoantibodies, Proulle et al. found that platelets, rather than endothelial cells, were the first effector cells activated by the anti-β2GPI antibody/β2GPI complex (37). Moreover, the anti-β2GPI antibody/β2GPI complex enhances platelet activation, and the secretion from activated platelets promotes endothelial cell activation (37). These findings underscore the critical role of platelets in the pathogenesis of APS.

#### 4.2.5 Procoagulant signaling in plasma membrane microdomains: focus on lipid rafts and heparanase

Although soluble coagulation factors and intracellular signaling pathways are well understood in APS pathogenesis, recent study showed that plasma membrane microdomains play an important role in regulating prothrombotic events. Specifically, lipid rafts and heparanase, despite working through different mechanisms, worsens the risk of thrombosis in APS.

Lipid rafts are believed to play an important role in the development of APS. They are small (10-200 nm) non-homogeneous regions of the membrane, rich in glycosphingolipids and cholesterol (170). In APS, studies have shown that anti-β2GPI antibodies bind to their target antigens, such as β2GPI, annexin A2, TLR2, and TLR4, within lipid rafts in the plasma membrane of monocytes or endothelial cells (133). Biochemical analyses revealed that β2GPI exists in a dimeric form within lipid rafts. This suggests that β2GPI can only interact with lipid rafts after it dimerizes, possibly due to conformational changes (171). Further studies showed that lipid rafts play a key role in the signaling pathway triggered by anti-β2GPI antibodies. These antibodies activate IRAK and NF-κB through lipid rafts, leading to the release of TNF-α and TF, which contribute to a proinflammatory and procoagulant state (172, 173). Additionally, it has been found that anti-β2GPI antibodies activate LRP6 and LRP8/ApoER2 signaling in endothelial cells through lipid rafts (173, 174). Riitano et al. used a raft-disrupting agent called methyl-β-cyclodextrin (MβCD) to further study the role of lipid rafts. The results revealed that anti-β2GPI antibodies induce TF expression in endothelial cells through the LRP8/ApoER2 signaling pathway, which requires intact lipid rafts for efficient signal transduction (174). These findings underline the importance of lipid rafts in APS and suggest potential targets for therapeutic intervention in APS.

Heparanase is the only known enzyme that cleaves heparan sulfate side chains, contributing to inflammatory disorders by aiding vascular endothelial cell migration and immune cell activation (175). It affects coagulation by upregulating TF expression and interacting with TFPI, increasing coagulation activity (176). Heparanase also acts as a cofactor for TF, promoting factor Xa production (177). In APS, inhibiting heparanase, such as RDS3337, prevents TF expression and platelet aggregation (175), suggesting that targeting heparanase could be a potential treatment for APS-related prothrombotic conditions.

### 4.3 Complement activation

Given the established interactions between the complement and the coagulation systems, complement components may be directly involved in thrombosis (178). Supporting this view, injecting IgG aPL purified from APS patients into complement C6-/- rats does not induce thrombosis in the mesenteric blood vessels but does cause blood clots in normal rats (57). A subsequent study replicated these findings in C6 knockout mice (179). Activation of complement components C3 and C5 can enhance aPL-mediated thrombosis and activate endothelial cells (28). APL-induced complement activation generates downstream C5a, which recruits and activates neutrophils, resulting in TF expression (180, 181). These animal experiments confirmed the critical role of complement in aPL-mediated thrombosis. Several studies have shown significantly elevated levels of complement activation products (fragments Bb and C3a-desArg) in patients with APS, which correlate with increased aPL titers (182, 183). Nevertheless, the underlying mechanisms driving complement activation in patients with APS are not yet fully understood. Recent study indicated that reduced levels of complement factor H (FH), a key regulatory factor, may contribute to complement activation in APS (184). A prior study used ELISA to detected FH autoantibodies in APS patients from Serbian and Italian cohorts, suggesting these autoantibodies may contribute to reduced FH levels (185). In 2016, a case report provided evidence that complement directly impacted human thrombosis. The report described an APS patient undergo femoral artery bypass surgery for vascular thrombosis, with complement deposition observed on the endothelium and vascular wall at the thrombotic site (186). Treatment with a humanized anti-C5a monoclonal antibody (eculizumab) effectively prevented thrombosis following the surgery (186). Furthermore, FXa and thrombin can activate the complement pathway by cleaving C3 and C5. However, β2GPI inhibits complement activation by modulating the activities of thrombin and FXa (187, 188). This interplay between coagulation factors and β2GPI may indicate a potential mechanism for complement activation.

Complement activation is considered a significant factor in early pregnancy loss (189, 190). Shamonki et al. conducted an immunohistochemical analysis using antibodies against C4d, C3b, and C5b-9 on placental tissue from APS patients and controls, highlighting complement’s crucial role in APS-related fetal tissue damage (191). Elevated levels of Bb and sC5b-9 detected in early pregnancy have been strongly associated with negative pregnancy outcomes in patients with aPL (192). Studies indicated that inhibiting the complement cascade with C3 convertase inhibitors or blocking C5a receptor interactions could mitigate the harmful effects of aPL in early pregnancy (193). Further evidence showed that heparins alleviated early pregnancy complications mainly by inhibiting aPL-induced complement activation, rather than through their anticoagulant properties (194). Genetic mutations that cause immune dysregulation are associated with complement-mediated diseases (195), and approximately 60% of catastrophic APS patients have germline variants in complement regulatory genes (196).

## 5 Two-hit model

**Figure 2:**
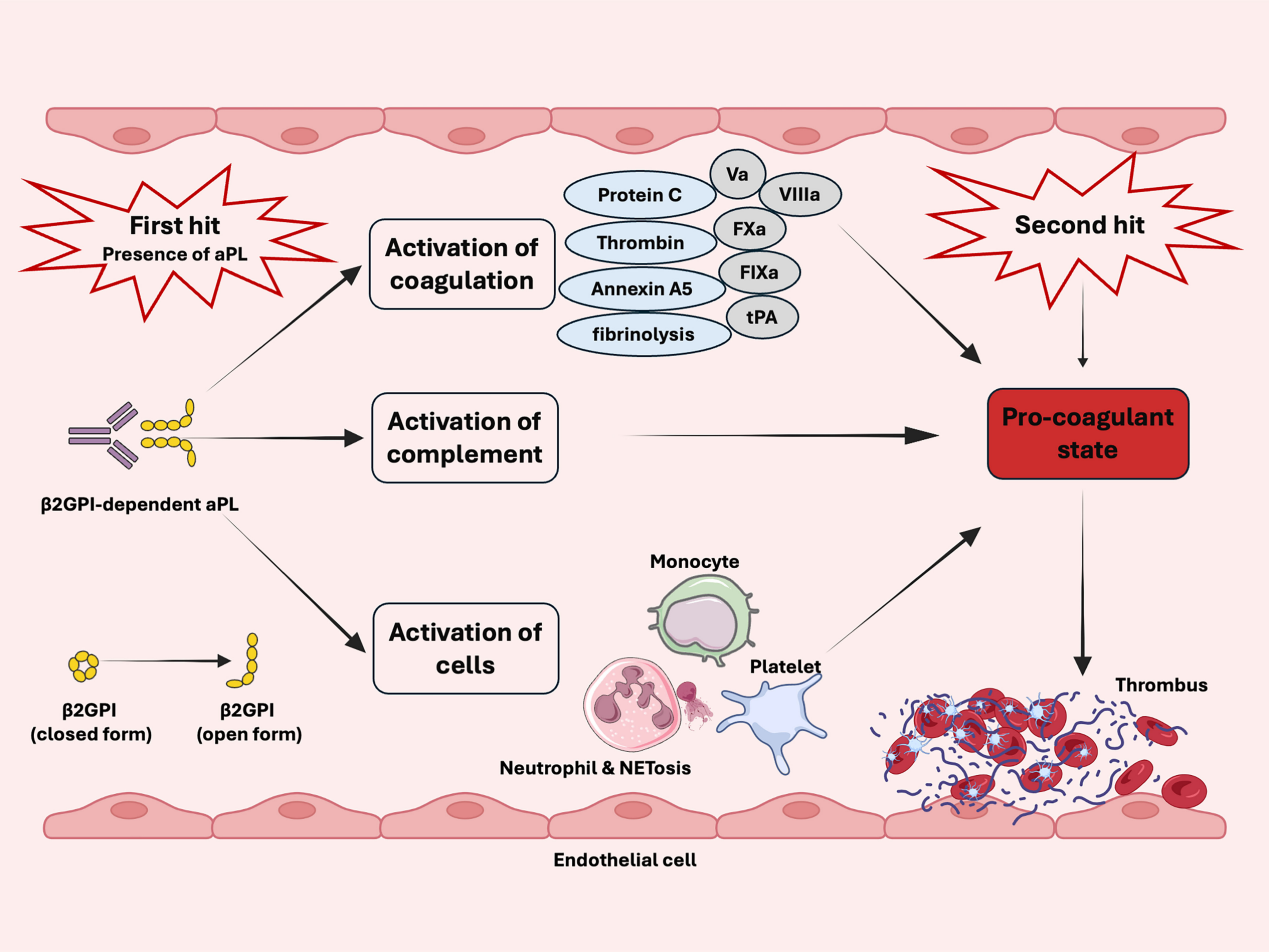
Schematic diagram of two-hit model in antiphospholipid syndrome. The presence of aPL (first hit) and the activation of the coagulation system, complement, monocytes, endothelial cells, neutrophils, and platelets increase the risk of thrombosis. Clotting occurs in the presence of additional procoagulant condition (second hit).

Thrombotic events are infrequent, even with the persistent presence of aPL. Moreover, aPL alone do not seem to induce thrombotic phenotypes (197). The ‘two hit’ hypothesis, proposed in 2001, explains these clinical and experimental observations (198). The presence of aPL (the first hit) increases the risk of thrombophilia, while clotting occurs when an additional procoagulant condition (second hit) is present (**Figure 2**) (198). The ‘two-hit’ model was later validated using a photochemically induced thrombosis model in hamsters (33). A low dose of bacterial lipopolysaccharide was necessary for β2GPI-dependent aPL to trigger thrombosis in rat mesenteric microcirculation (57). Consistent with this finding, infection might act as a second hit, amplifying the thrombophilic effect of aPL (199).

The presence of aPL is recognized as necessary but insufficient from thrombosis in APS while an additional ‘second hit’ is required (18, 22, 23, 47, 72, 200). The second hit often includes but not limited to inflammatory responses, mechanical trauma, immobility, venous stasis and estrogen-containing contraceptive (22, 47, 72). In addition, cardiovascular risk factors, such as arterial hypertension, diabetes, obesity, smoking, and hyperlipidemia, further increase the risk of thrombosis (72, 200).

## 6 Current and potential drugs for APS

The management of thrombotic APS focuses on preventing thrombotic events and addressing the multifaceted pathogenesis of the disease. Current strategies emphasize anticoagulation as the cornerstone of therapy, targeting the hypercoagulable state driven by aPL. Standard regimens typically include vitamin K antagonists and low-molecular-weight heparin (LMWH), with close monitoring of the international normalized ratio (INR) to ensure an appropriate balance between thromboprophylaxis and the risk of bleeding. Immunomodulation plays a critical role in suppressing autoimmune-driven pathways, particularly in patients with concurrent SLE or refractory cases. **Table 1** summarizes the primary therapeutic agents for preventing thrombotic events in APS.

**Table 1:** Drugs used in the management of APS.

Drug classes	Medication	Key mechanism	Perspective
Vitamin K antagonist	Warfarin	Inhibits factors II/VII/IX/X (201)	Standard regimen for thrombotic APS (202)
Anticoagulant	LMWH	Inhibits Xa/IIa (201)	In acute thrombosis, obstetric APS (203)
Direct oral anticoagulant	Rivaroxaban	Inhibits Xa/IIa (201)	Rivaroxaban had a higher risk of recurrent arterial thrombosis compared to warfarin (204)
Antiplatelet agent	Aspirin	Blocks COX-1/TXA2 (205)	Primary prophylaxis in high-risk APS (206)
Antimalarial	Hydroxychloroquine	Reduces cytokines, inhibits complement activation, suppresses TLR4 (207)	In SLE-associated APS, pregnancy (206)
Complement inhibition	Eculizumab	Neutralizes C5a/MAC	In catastrophic APS (208, 209)
Triple Therapy	Heparin& glucocorticoids& intravenous immunoglobulin	Neutralizes aPL, anticoagulates, suppresses inflammation (210)	In life-threatening catastrophic APS (202)
B cell therapy	Rituximab	Type I anti-CD20 monoclonal antibody, depletes aPL-producing B cells (211)	In refractory APS, catastrophic APS (202)
	Obinutuzumab	Type II anti-CD20 monoclonal antibody, depletes aPL-producing B cells (211)	Alternative option for rituximab in APS (212)
	Belimumab	Inhibits B-lymphocyte stimulator, reducing autoreactive B-cell activation (213)	In SLE-associated APS (especially lupus nephritis), primary APS with high thrombotic risk (213, 214)
mTOR inhibition	Sirolimus	Inhibits mTOR signaling pathway, reduces the proliferation of endothelial cells (215)	APS-related vascular lesions (e.g., nephropathy), refractory thrombotic APS (216, 217)
Cytotoxic drug	Cyclophosphamide	Suppresses B/T-cell proliferation and autoantibody production (218)	In severe SLE-associated APS organ involvement (219, 220)
NETs Inhibitors	Defibrotide	Inhibits NETosis via cAMP signaling, inhibits endothelial activation (135, 221)	APS-associated hepatic venous thrombosis, microvascular thrombotic complications (221, 222)

While anticoagulants remain foundational, their inability to prevent non-thrombotic complications underlines the need for potential therapies. Potential approaches aim to tackle non-thrombotic complications and specific pathogenic mechanisms that lead diseases. Among these strategies, statins have shown dual benefits by not only improving endothelial function through lipid-lowering effects but also suppressing aPL-induced pro-inflammatory signaling (e.g., NF-κB and MAPK pathways), thereby reducing TF expression and monocyte activation (223). Direct targeting of coagulation activation is exemplified by anti-TF molecules (e.g., ALT-836), which inhibit thrombin generation by neutralizing TF upregulated in monocytes (224). In addition, interventions against NETs, such as DNase-mediated degradation, PAD4 inhibition, and histone toxicity blockade, aim to mitigate NETs-driven thromboinflammation and placental damage (225). To disrupt the core antigen-antibody interaction in APS, anti-β2GPI domain I monoclonal antibodies competitively block pathogenic aPL binding to β2GPI, preventing its engagement with cellular receptors like ApoER2 and subsequent complement activation (226, 227). Further upstream, lipid raft-targeting agents (e.g., MβCD) destabilize membrane microdomains essential for β2GPI anchoring, thereby suppressing platelet activation and complement deposition (173). Beyond thrombotic pathways, immune modulation is being explored through probiotics and vitamin D3, which restore gut microbiota balance, regulate Th17/Treg dynamics, and suppress autoreactive B-cell responses (202, 228, 229). Collectively, these therapies exemplify a paradigm shift from broad anticoagulation to mechanistically precise interventions targeting aPL-mediated thrombosis, autoantibody pathogenicity, and immune dysregulation.

## 7 Conclusion and future directions

Despite the strong association between aPL and thrombosis, the exact mechanisms underlying aPL-mediated thrombotic events remain unclear. Current evidence suggests that aPL triggers activation of endothelial cells, monocytes, neutrophils, and platelets. This activation, coupled with the disruption of natural anticoagulant and fibrinolytic pathways, leads to a pro-thrombotic state in patients with thrombotic APS. An additional triggering event, or ‘second hit,’ is ultimately necessary to initiate thrombus formation.

Despite extensive research efforts, several crucial questions remain unanswered. One unresolved question is which cell type serves as the primary target for aPL. Some studies argue that endothelial cells play a key role in APS-associated thrombosis (104), while others downplay the role of the endothelium and emphasize the importance of platelets (37). Recent insights suggest that anti-inflammatory treatments targeting NETosis may be more effective than conventional anticoagulation therapy in reducing thrombosis (230). It is also possible that all of these cell types contribute, either directly or indirectly, through the release of prothrombotic microparticles (231, 232). While it is well-established that a ‘second hit,’ such as mechanical trauma combined with antibody binding to β2GPI on the endothelium, plays a key role in initiating clot formation at specific sites (18), it remains obscure how other types of ‘second hit’ trigger thrombosis. In addition, β2GPI, the primary antibody target, is a complex protein with an unclear physiological role (62). Understanding the precise function of β2GPI could be crucial for unraveling the mechanism of thrombosis. Finally, a consensus on the optimal animal model that accurately replicates APS pathophysiology is urgently needed to improve *in vivo* experiments. Advances in APS research will deepen our understanding of the underlying mechanisms and contribute to future treatments.
